# N-Acetylcysteine Promotes Metastatic Spread of Melanoma in Mice

**DOI:** 10.3390/cancers14153614

**Published:** 2022-07-25

**Authors:** Elena Obrador, Rosario Salvador-Palmer, Rafael López-Blanch, María Oriol-Caballo, Paz Moreno-Murciano, José M. Estrela

**Affiliations:** 1 Department of Physiology, Faculty of Medicine and Odontology, University of Valencia, 46010 Valencia, Spain; rosario.salvador@uv.es (R.S.-P.); loblanch@alumni.uv.es (R.L.-B.); maria.oriol@uv.es (M.O.-C.); 2 Scientia BioTech S.L., 46002 Valencia, Spain; paz.moreno72@gmail.com; 3 Department of Physiology, Faculty of Pharmacy, University of Valencia, 46100 Burjassot, Spain

**Keywords:** melanoma, metastases, N-acetylcysteine, cyst(e)ine, glutathione, thiols, oxidative stress

## Abstract

**Simple Summary:**

Malignant melanoma is a cancer derived from melanocytes, the cells that produce pigment (melanin) in the skin. It develops on the skin, but can also appear on the mucous membranes and in other locations. Melanomas are responsible for 80% of deaths related to skin cancers. In recent years, the number of cases has increased alarmingly, likely in relation to sun exposure habits. Once melanoma spreads to distant parts of the body, the 5-year survival rate is about 10%. N-acetylcysteine (NAC) is a drug with antioxidant properties, and thereby could play a role in preventing cancer. NAC is commonly used as a mucolytic in different respiratory diseases, to treat acetaminophen (Tylenol) poisoning, and is also present in different nutritional supplements. Nevertheless, the use of NAC and other antioxidants in cancer has been questioned. Here, we show that high therapeutic doses of NAC may cause metastatic spread of a malignant melanoma.

**Abstract:**

N-acetylcysteine (NAC) is a direct Cys donor and a promoter of glutathione (GSH) synthesis. GSH regulates melanoma growth and NAC has been suggested to increase melanoma metastases in mice. We found that high therapeutic doses of NAC do not increase the growth of melanoma xenografts, but can cause metastatic spread and distant metastases. Nevertheless, this is not due to an antioxidant effect since NAC, in fact, increases the generation of reactive oxygen species in the growing metastatic melanoma. Trolox, an antioxidant vitamin E derivative, administered in vivo, decreased metastatic growth. Metastatic cells isolated from NAC-treated mice showed an increase in the nuclear translocation of Nrf2, as compared to controls. Nrf2, a master regulator of the antioxidant response, controls the expression of different antioxidant enzymes and of the γ-glutamylcysteine ligase (the rate-limiting step in GSH synthesis). Cystine uptake through the xCT cystine-glutamate antiporter (generating intracellular Cys) and the γ-glutamylcysteine ligase activity are key to control metastatic growth. This is associated to an increase in the utilization of L-Gln by the metastatic cells, another metastases promoter. Our results demonstrate the potential of NAC as an inducer of melanoma metastases spread, and suggest that caution should be taken when administering GSH promoters to cancer patients.

## 1. Introduction

Glutathione (GSH, γ-L-glutamyl-L-cystenyl-glycine) is the most prevalent, non-protein thiol in mammalian cells [1]. There is an evident correlation between high proliferation activity and high GSH levels in melanoma cells, a fact which was first detected by our group [2]. Moreover, high GSH levels also associate with high metastatic activity [3]. Consequently, agents that can lead to an increase in cancer GSH levels may also promote their growth and dissemination.

The rate-limiting steps for GSH synthesis are the activity of the γ-glutamyl-cysteine ligase (GCL), the first enzyme in the GSH biosynthesis, and the availability of Cys [4]. Free Cys levels, both within cells and extracellularly, are very low [5,6]. Thus, any source of free Cys could potentially favor the growth of a melanoma.

A research work published in 2015 [7] suggested that antioxidants, including N-acetylcysteine (NAC) and the soluble vitamin E analog Trolox, can increase melanoma metastasis in mice. However, other previous communications suggested just the opposite [8,9,10,11,12,13,14]. However, and contributing to the controversy, other studies did support a protumoral effect of NAC [15,16].

NAC is a classical drug used as a mucolytic or to treat acetaminophen overdose, and is also used as a nutritional supplement, in combination with other molecules (e.g., Celltrient^TM^, Nestlé Health Science) [17]. Nevertheless, reports showing e.g., that NAC treatment protected from lung emphysema, but concomitantly induced the development of lung adenocarcinoma in mice [18], are cause for concern. Thus, taking into account what has been said about its relationship with GSH, and the metastatic activity of melanoma and other cancers, the need to clarify what effect it may have is evident, if this effect is dose dependent, and if it is related to the in vivo levels of Cys and/or GSH. In the present contribution, we have analyzed this problem using murine and human malignant melanomas.

## 2. Materials and Methods

### 2.1. Culture of Melanoma Cells and Keratinocytes

Human BRAFV^600E^ (A2058, COLO-679, and SK-Mel-28) and murine B16F10 melanoma cells were from the ATCC [18] (Manassas, VA, USA). Melanoma were grown in DMEM (Invitrogen, San Diego, CA, USA), pH 7.4, supplemented with 10% heat-inactivated FCS (Biochrom KG, Berlin, Germany), 100 units/mL penicillin and 100 μg/mL streptomycin. Cells were plated (20,000 cells/cm^2^) and cultured at 37 °C in a humidified atmosphere with 5% CO_2_. Cells were harvested by incubation for 5 min with 0.05% (*w*/*v*) trypsin (Sigma Aldrich, St. Louis, MO, USA) in PBS, pH 7.4, containing 0.3 mM EDTA, followed by the addition of 10% FCS to inactivate the trypsin. Cells were allowed to attach for 12 h before any treatment addition.

Primary mouse keratinocyte were isolated from the skin and cultured as described by [19].

Cell number and trypan blue exclusion-based viability were determined using a BioRad (Hercules, CA, USA) TC20 Automated Cell Counter. Cell integrity was also confirmed by measuring leakage of lactate dehydrogenase activity.

### 2.2. Mice and Orthotopic Xenografts

Nude (nu/nu) mice (male, 9–10 weeks old) and syngenic male C57BL/6J mice (male, 12 weeks old (Charles River Laboratories, Wilmington, MA, USA), were fed ad libitum on a standard diet (Letica, Rochester Hills, MI, USA), and kept on a 12-h-light/12-h-dark cycle with the room temperature at 22 °C. To generate orthotopic xenografts, mice were inoculated intradermally (on the back) with 2 × 10^6^ melanoma cells per mouse. Tumor volume was measured using calipers and expressed in mm^3^ according to V = 0.5a × b^2^ (a and b are the long and short diameters, respectively). Xenografted tumors were allowed to grow for a maximum of 3 weeks and then were surgically removed. For histological analysis, skin tumors were fixed in 4% formaldehyde in PBS (pH, 7.4) for 24 h at 4 °C, paraffin embedded, and stained with hematoxilin and eosin and safran. The sacrifice was performed by cervical dislocation.

### 2.3. NAC Administration to Tumor-Bearing Mice

Each dose of NAC was administered orally (dissolved in tap water and having the pH adjusted to 7.4 with NaOH). In humans, it is most commonly taken by mouth in doses of 600–1200 mg daily (e.g., www.webmd.com (accessed on 15 June 2021)). We used the conversion factor (12.3) recommended by the FDA to convert human doses (assuming a 60 kg human) into animal (mice) doses (www.fda.gov/media/72309/download (accessed on 15 June 2021)). Thereby, 10–20 mg NAC/kg human corresponds to approx. 120–240 mg/kg mouse. The total dose of NAC per day was divided into two identical administrations, one every 12 h.

### 2.4. Determination of NAC, Cys and Cystine Levels in Plasma

Blood samples were collected in sodium citrate tubes at 4 °C. Blood plasma was obtained by low-speed centrifugation (10 min at 1000× *g*). Reversed-phase high-pressure liquid chromatography was based on a previously described methodology [20]. For cystine determination, plasma protein was precipitated with 3.75% (*w*/*v*) sulphosalicylic acid in 0.3 M-lithium (1:4), and a sample of the supernatant was analyzed in an LA8080 high-speed amino-acid analyzer (Hitachi, Tokyo, Japan).

### 2.5. Isolation of Melanoma Cells Using Enzymatic Digestion and a Double Ficoll Gradient

To maximize cell yield and viability we used collagenase III (200 U/mL; Sigma-Aldrich, St. Louis, MO, USA), DNase I (200 U/mL; Sigma-Aldrich) and trypsin (5 mg/mL; Invitrogen, Waltham, MA, USA), and a non-enzymatic dissociation buffer (NEDB, Invitrogen). The excision of the tumors, their subsequent processing and the isolation of the cancer cells, was carried out following a procedure previously described in detail [21].

### 2.6. Experimental Metastases

Hepatic metastases were generated by i.v. injection (portal vein) into anesthetized mice (Nembutal, 50 mg/kg i.p.) of 105 viable B16-F10 melanoma cells, suspended in 0.2 mL of DMEM. Mice were cervically dislocated 10 days after tumor cell inoculation. Livers were fixed with 4% formaldehyde in PBS (pH 7.4) for 24 h at 4 °C and then paraffin embedded. Metastasis volume (mean percentage of organ volume occupied by metastases) was determined as described previously [3].

### 2.7. Enzyme Assays

γ-glutamyl transpeptidase (GGT), superoxide dismutase (SOD) 1 and 2, catalase (CAT) and glutathione peroxidase (GPX) activities were measured as previously described in detail [22].

### 2.8. RT-PCR and Detection of mRNA

RNA was extracted using Qiagen RNAeasy mini kits (Hilden, Germany). Quantitative and qualitative analyses of RNA samples were performed using a 2100 Bioanalyzer (Agilent Technologies, Santa Clara, CA, USA). cDNA was obtained using a random hexamer primer and a MultiScribe reverse transcriptase kit, as recommended by the manufacturer (Taq-Man RT Reagents; Thermo Fisher Scientific, Waltham, MA, USA). PCR master mix and AmpliTaq Gold DNA polymerase were added to the specific primers (Sigma Genosys, Haverhill, UK), as previously reported, for GCL (catalytic subunit), [nuclear factor (erythroid-derived 2)-like 2] (Nrf2) and glucose-6-P dehydrogenase (G6PDH) [23]. Real-time quantification of mRNA relative to G6PDH) was performed as previously reported [21]. The NE-PER extraction kit from Thermo Fisher Scientific was used for nuclear protein extraction, according to the manufacturer’s instructions.

### 2.9. Western Blots

Western blot analysis was performed as previously described [24]. Protein bands were quantified using laser densitometry.

### 2.10. GSH and GSSG Determination

GSH and GSSG (glutathione disulfide) were determined by LC/MS as previously reported [23].

### 2.11. Knockdown of GCLC, SLC7A10 and SLC7A11genes

Knockdown of GCLC (codes the γ-glutamate-cysteine ligase catalytic subunit) by small hairpin RNA (shRNA) was performed following the methodology described by Díaz-Hernandez et al. [25]. Knockdown of SLC7A10 (codes the ASC1 Na^+^-independent neutral amino acid transporter) was performed using an ASC1 shRNA Plasmid sc-39159-SH from Santa Cruz Biotechnology (Dallas, TX, USA). Knockdown of SLC7A11 (codes the xCT Na^+^-independent cystine-glutamate antiporter) by shRNA was based on the methodology described by Lastro et al. [26]. Scrambled sequences were used as controls.

### 2.12. Analysis of Amino Acids

Levels of amino acids in arterial blood were determined as previously described [22] and using an LA8080 high-speed amino-acid analyzer (Hitachi, Tokyo, Japan).

### 2.13. Rate of Cyst(e)ine Uptake

Rates of Cys and cystine uptake by the melanoma cells were calculated after administering i.v. 2.0 μCi of [^35^S]Cys or 10.0 μCi of [^35^S]cystine (PerkinElmer, Waltham, MA, USA).

### 2.14. Rates of Glucose and Glutamine Utilization

These rates were measured as previously described [27].

### 2.15. H_2_O_2_ and O_2_^·−^ Generation Assays

Quantitative measurement of H_2_O_2_ and O_2_^·^^−^ generation followed previously described methodology [22].

### 2.16. Rate of Oxygen Utilization

O_2_ concentration and consumption in isolated melanoma cells were measured using an oxygraph of OROBOROS Instruments (Innsbruck, Austria) and as previously described [22].

### 2.17. Statistics

Data are presented as mean values ± SD for the number of different experiments. Statistical analyses were performed (unless specifically indicated) using Student’s *t*-test, and *p* < 0.05 was considered significant.

## 3. Results

### 3.1. NAC Supplementation Does Not Increase the Growth of Melanoma Xenografts

**Figure 1:**
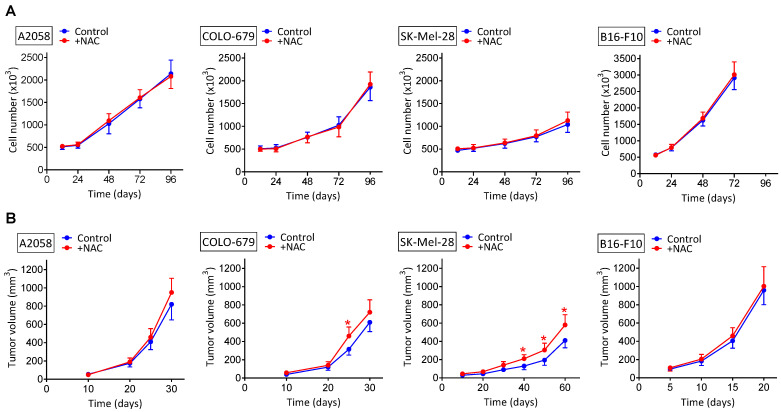
Effect of NAC on the rate of melanoma growth in vitro and in vivo. (**A**) NAC (2 mM) was added to the culture medium 24 h and 72 h after seeding. All data are mean values ± SD of 5–6 different experiments. No significant differences were found comparing NAC-treated cells versus controls. (**B**) NAC (240 mg/kg × day) was administered for 2 weeks, starting on day 14 in mice bearing A2058 or COLO-679 melanoma, on day 21 in mice bearing the SK-Mel-28 melanoma, and on day 5 in mice bearing the murine B16-F10 melanoma. All data are mean values + SD (n = 15 mice). * Significantly different *p* < 0.05, comparing NAC-treated mice versus controls treated with vehicle.

We tested to see if NAC administration affects in vitro and/or in vivo (human and murine) melanoma growth. As shown in Figure 1A, in vitro melanoma cell growth was not significantly affected, as compared to controls, by adding to the cultured medium NAC at a high concentration (2 mM). The maximum in vivo dose indicated above under NAC administration to tumor-bearing mice, 240 mg/kg, if distributed rapidly and homogenously in the mouse water (70% of the total weight), would reach a concentration of approx. 2 mM. Therefore, the concentration we used under in vitro conditions reflects the maximum concentration to which melanoma cells could be exposed in vivo.

Melanoma-bearing mice were treated with NAC at three different dosages (30, 120 and 240 mg/kg × day, where each total dose was divided in two identical doses given every 12 h) (Figure 1B). This dosage schedule reflects current recommendations. Therefore, a low dose of approx. 30 mg/kg × day, relevant from a nutraceutical point of view, was compared with two pharmacological doses (120 and 240 mg/kg × day). Neither the lowest dose (30 mg/kg) nor the 120 mg NAC/Kg affected the rate of melanoma growth, as compared to controls (not shown). Only the highest dose (240 mg/kg) showed a tendency to increase the melanoma growth, but this was only statistically significant in the SK-Mel-28 model (Figure 1B).

### 3.2. NAC Supplementation Promotes Metastatic Spread despite Surgical Removal of Primary Growing Melanoma Xenografts

**Figure 2:**
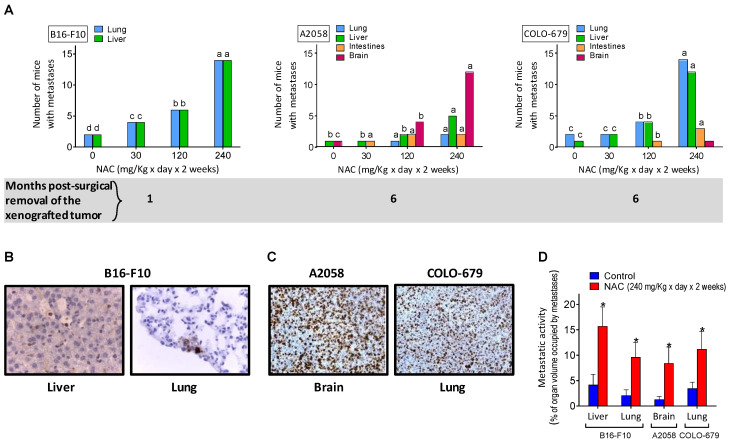
NAC administration promotes metastatic spread in murine and human melanomas. Independently of the dose, NAC was administered as indicated in Figure 1B. All melanoma cell lines described in this Figure were inoculated intradermally (on the back) with 2 × 10^6^ melanoma cells per mouse as indicated in the Materials and Methods section. Melanoma xenografts were surgically removed on days 30 (A2058 and COLO-679), 60 (SK-Mel-28), and 20 (B16-F10) post-inoculation. Mice were sacrificed at 6 months (human melanomas) or 1 month (B16-B10), after removal of the primary tumor. The presence of metastatic foci was investigated in four different organs, which are classical sites for melanoma metastases. (**A**) Number of mice (n = 20 in each group) where growing metastases were found. No metastases were found in mice that were initially inoculated with the SK-Mel-28 melanoma. A one-way analysis of variance (ANOVA) was used to make comparisons among the different treatment groups. Different superscript letters indicate differences for each organ where metastases where found, *p* < 0.05. (**B**) Microscopic images showing B16-F10 melanoma cells found in the liver and lung (immunochemical detection using S100 monoclonal antibodies) of mice pretreated with NAC. (**C**) Cell proliferation in metastases (A2058 in the brain and COLO-679 in the lung of mice pretreated with NAC) using anti-Ki-67 monoclonal antibodies. (**D**) The percentage of organ volume occupied by metastases (**A**) was analyzed following the methodology indicated under Materials and Methods. * Significantly different *p* < 0.01, comparing NAC (240 mg/Kg × day × 2 weeks)-treated mice versus untreated controls.

The rapid growth rate of intradermally inoculated melanomas (Figure 1B) does not allow the detection of possible disseminated metastases. Thus, to avoid sacrificing animals for ethical reasons linked to the size of the tumor, we removed the tumor xenografts surgically and searched if, over time, some of the melanomas had generated metastatic spread. As shown in Figure 2A, one month after the B16-F10 removal, just a few mice (two out of a group of 20) showed lung and liver metastases. However, pharmacological doses of NAC, administered for 2 weeks before tumor removal, significantly increased the number of mice showing B16-F10-derived metastases (Figure 2A), and also the % of organ volume occupied by metastases (Figure 2D). The 2-week protocol of NAC administration (see NAC administration to tumor-bearing mice under Material and Methods) is standard in many clinical indications where NAC is utilized [17]. NAC-induced metastatic spread was also detected in two of the human melanoma models studied (A2058 and COLO-679) (Figure 2B). NAC was administered, starting on day 14 in mice bearing A2058 or COLO-679 melanoma, on day 21 in mice bearing the SK-Mel-28 melanoma, and on day 5 in mice bearing the murine B16-F10 melanoma. Although all models were treated with NAC for 2 weeks, the reason for the differences in the start of administration lies in the different rates of progression of the xenografts. The common factor in all cases was to start the administration approximately when the exponential phase of growth begins in each case. All these facts prompted us to investigate the underlying mechanisms and, specifically, if Cys acts as a metastasis’s promoter.

### 3.3. shRNA-Induced Downregulation of Cystine Uptake or γ-GCS Activity Decreases Metastatic Melanoma Growth

**Figure 3:**
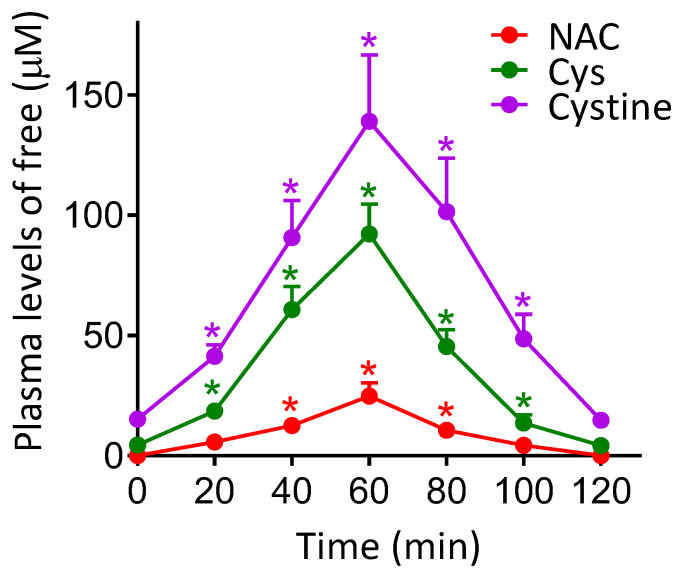
Effect of NAC administration on the plasma levels of free NAC, Cys and cystine. NAC (240 mg/kg) was administered orally to mice bearing B16-F10 xenografts starting 5 days after tumor inoculation. NAC, Cys and cysteine levels were determined by reversed-phase high-pressure liquid chromatography, as explained under Materials and Methods, 15 days after tumor inoculation. Data are mean values ± SD, n = 5–6 determinations per molecule and time point. * Significantly different *p* < 0.01, comparing data for NAC at 40–100 min versus NAC levels at 20 min; comparing data for Cys and cysteine levels at 20–120 min versus control levels of Cys and cysteine at 0 min (n = 7 in each case).

As shown in Figure 3, NAC administration (240 mg/kg) to B16-F10-bearing mice induces a significant increase in the circulating levels of Cys and cystine. Peak values were detected 60 min after NAC administration (approx. 90 μM Cys and 140 μM cystine in plasma). These data were not significantly different if NAC was administered to mice bearing A2058 or COLO-679 xenografts (not shown). This experimental evidence raised the question of a possible relationship between the increase in bioavailable levels of cyst(e)ine and, eventually, the increase in metastatic spread.

In this regard, the B16-F10 model is characterized by its high metastatic potential. Moreover, its intravascular inoculation and preference for metastatic colonization in the liver and lungs is a well-established methodology. Thus, we focused on this model for our further studies.

As indicated in the Introduction, high GSH levels in melanoma cells associate to high metastatic activity. Since Cys is a rate-limiting precursor for GSH synthesis, it seems plausible that there is a possible link between the increase in bioavailable cyst(e)ine levels and the GSH content in the melanoma cells. To further investigate this, we first measured the effect of NAC administration on the concentration of precursor amino acids for GSH synthesis in the plasma of mice bearing B16-F10 xenografts. As shown in Table 1A, in B16-F10-bearing xenografts, treatment with NAC slightly decreased the plasma levels of Gln and Met, whereas it increased by 10-fold the levels of cyst(e)ine.

In addition, we also measured the effect of NAC administration on the GGT activity and the rates of Cys and cystine uptake in B16-F10 melanoma cells isolated from tumor-bearing mice (15 days after in vivo inoculation). GGT degrades extracellular GSH (that exported from organs/tissues–mainly the liver- and transported by the blood circulation), thus releasing more free Cys [28]. As shown in Table 1B, NAC treatment did not affect the GGT activity. Therefore, an increase in Cys release from extracellular GSH (catalyzed by GGT) is not responsible for the NAC-induced increase in Cys uptake by the melanoma cells. However, NAC treatment increases the plasma levels of Cys and cystine (Figure 3), and associates to a two-fold increase in the uptake of both amino acids by the melanoma cells (Table 1B); an effect that may lead to an increase in the levels of GSH in melanoma cells. Indeed, we found that control B16-F10 melanoma cells growing as xenografts have a GSH content of 26 ± 4 nmol/10^6^ cells on day 15 after inoculation, and of 40 ± 5 nmol/10^6^ cells on the same day, but after mice were previously treated with NAC (240 mg/kg × day) for 10 days (*n* = 7 in both cases, * *p* < 0.01 comparing NAC-treated mice versus controls).

Our next step forward was to investigate how Cys and cystine supply, or GSH synthesis and levels, affect metastatic activity. For this purpose we use specific shRNAs to knockout the alanine-serine-cysteine transporter ASC1 transporter (the main system for Cys uptake) [29], the xCT cystine/glutamate antiporter (the main system for cystine uptake) [30], and the GCLC (the rate-limiting enzyme in the synthesis of GSH) [4] (see Supplementary Figure S1). As shown in Table 2, GSH levels in the metastatic cells are highly dependent on cystine supply. Inhibition of the xCT-dependent cystine uptake decreased the metastatic growth in the liver by approx. 50%, either in non-treated mice or in NAC-treated mice (Table 2). Cystine uptake in nmol/mg protein × min is much higher than that of Cys (Table 1B). In consequence to this fact, it is the inhibition of the xCT cystine/glutamate antiporter (the main system for cystine uptake) [29], and not the inhibition of Cys uptake, that causes a significant decrease in GSH levels and the metastatic activity (Table 2). Importantly, knockout of the rate-limiting enzyme for the GSH synthesis further decreased metastatic growth to approx. 25% of the control values. This fact highlights further the importance of GSH in the control of metastatic growth.

Furthermore, we also found that NAC treatment associates with a decrease in the rate of glucose utilization, and an increase in the rate of L-Gln utilization (Table S1). Both, glucose and L-Gln, are preferential sources of energy for the cancer cells [31]. In many cancer cells, including melanoma cells, L-Gln is the primary mitochondrial substrate and is required for maintenance of mitochondrial membrane potential and integrity, and for support of the NADPH production [32]. This increase in L-Gln consumption will render more glutamate to be used for GSH synthesis and the xCT cystine/glutamate antiporter.

### 3.4. Melanoma Metastases Show Higher ROS Generation in Mice Treated with NAC

**Figure 4:**
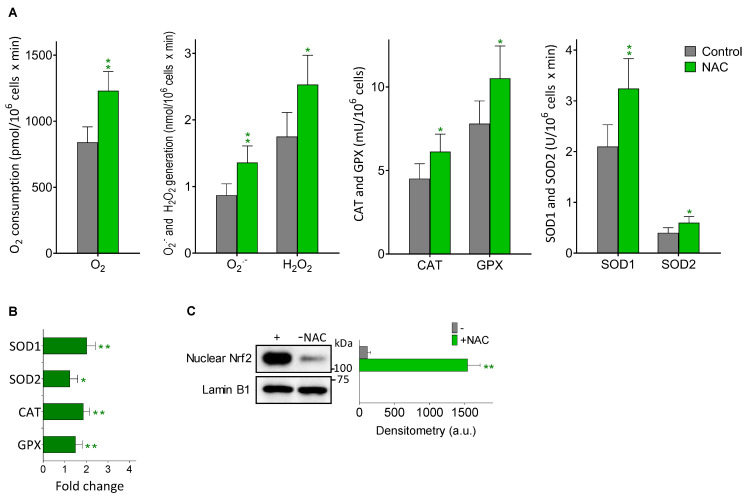
Effect of NAC treatment on the generation of ROS in metastatic B16-F10 cells. Melanoma cells were inoculated, mice treated, and metastatic cells isolated as in Table 2. (**A**) O_2_ consumption, ROS generation and oxidative stress-related enzyme activities (n = 5–6). (**B**) Expression of oxidative stress-related enzymes. Data express fold change (quantitative RT-PCR) (n = 5–6) comparing metastatic cells from NAC-treated mice versus controls. Significantly different * *p* < 0.05, ** *p* < 0.01. (**C**) Western blot analysis of the effect of NAC treatment on nuclear Nrf2. Densitometric analysis represents the mean values ± SD for three different mice and experimental condition (** *p* < 0.01, comparing NAC-treated mice versus controls).

Aggressive cancer cells, including melanoma cells, generate high basal levels of reactive oxygen species (ROS), compared to their normal counterparts [33,34]. ROS might contribute to the ability of metastatic melanoma cells to mutate and grow [35]. As NAC is an antioxidant, directly or by promoting the synthesis of GSH, one may expect that NAC treatment would reduce ROS generation in the metastatic melanoma cells. However, as shown in Figure 4A and as compared to controls, in metastatic B16-F10 cells isolated from the liver of NAC-treated mice, there is a significant increase in different parameters related to oxidative stress i.e., O_2_ consumption, O_2_^·^^−^ and H_2_O_2_ generation, and the activities of SOD1, SOD2, CAT and GPX. The increase in the four enzyme activities measured correlated with an increase in their expression (Figure 4B), and also with an increase in the nuclear translocation of Nrf2 (a master regulator of the anti-oxidative defense system [36]) (Figure 4C). Indeed, activation of NRF2 in melanomas, particularly in those harboring KEAP1 mutations, has been associated to during progression (see e.g., Carpenter et al. for a recent review [37]). As shown in Figure 4, it seems clear that NAC treatment induces a higher level of oxidative stress in the metastatic cells; an unfavorable situation that is compensated, at least in part, by the concomitant increase in antioxidant defenses. Nevertheless, it is possible that exposure to higher levels of internally generated ROS may favor the survival of highly resistant and aggressive metastatic cell subsets. Interestingly, NAC-induced oxidative stress in cancer cells has also been observed in e.g., leukemia cells [38].

It is also possible that metastatic melanoma cells may show organ-specific differences. In order to investigate this question, we ran some more experiments in B16-F10 cells isolated from lung metastases. To this end, B16-F10 cells were inoculated via the tail vein of the animals. We measured in these cells some key parameters that, in light of the findings obtained in isolated metastatic cells of the liver, can serve as comparisons. As shown in Table S2, and compared to the equivalent data obtained in metastatic cells in the liver (Table 1B), GGT, Cys uptake, and cystine uptake are quite similar. However, GSH levels and metastatic activity (Table 2) are significantly lower. Based on the relationship between GSH and metastatic activity, the fact that hepatocytes may serve as a paracrine source of GSH for the metastatic cells in the liver could explain the difference. GSH levels in the liver are higher than in the lung (see e.g., [39]). ROS generation (Figure 4) was also found to be slightly lower in the metastatic cells isolated from the lung (Table S2). All these facts together suggest that, indeed, there may be differences between organs.

Epidermal keratinocytes, the predominant cell type in the skin epidermis, are in the front line of skin defense. Ten days after melanoma removal, we also investigated the ROS generation status at the primary site (skin) where the tumors were surgically removed. To this end, keratinocytes were isolated from the skin and cultured. As shown in Table S3, we compared ROS generation in keratinocytes isolated from the skin of control non-tumor-bearing mice, of A2058 melanoma-bearing mice, and of mice where the A2058 melanoma was removed. ROS generation was found to be significantly higher in keratinocytes from melanoma-bearing mice, compared to controls. No significant difference was found between controls and mice where the tumor had been removed (Table S3).

## 4. Discussion

Despite the controversy on pro- or anti-tumoral effects of NAC raised by different authors (see the Introduction section), our results clearly demonstrate in different models that NAC does not increase melanoma growth, either in vitro or as primary xenografts (Figure 1A,B, respectively). Although we found a small NAC-induced increase in the growth of the SK-Mel-28 model, this is not enough to support a positive effect of NAC on the growth of primary melanomas. Nevertheless, these results do not rule out the possibility that NAC could induce a metastatic spread from the primary xenografted tumors, as suggested by the work of Le Gal et al. [7]. These authors showed that NAC and the soluble vitamin E analog, Trolox, increased the migration and invasive properties of human malignant melanoma cells, thus concluding that antioxidants can increase melanoma metastasis in mice [7]. In agreement with this seminal work, we found that, after surgical removal of the primary tumors, mice showed metastatic spread over time (Figure 2). This is also a known possibility affecting patients undergoing surgery to remove primary melanomas [40]. Importantly, we observed that NAC increased distant metastases in different (human and murine) models and in a dose-dependent manner (Figure 2). However, although Le Gal et al. [7] report that the antioxidants did not affect basal amounts of cellular ROS in melanoma cells, their data were not obtained in real metastatic cells. We found the opposite in isolated cells from in vivo growing metastases (Figure 4). This is a fundamental difference because, in fact, ROS might contribute to the metastatic potential and growth capacity of melanomas (e.g., [35,41]). A shown in Figure 4, NAC increased O_2_^·^^−^ and H_2_O_2_ generation, as part of a wider effect also involving different antioxidant enzymes. Consequently, our results compromise the statement that antioxidants (compounds that counteract the effect of ROS) can increase melanoma metastasis [7]. This is conceptually misleading. One issue is that NAC, or an equivalent thiol compound, may lead to an increase in metastatic spread and/or tumor growth. But an entirely different concept is that an antioxidant, which basically decreases oxidative damages, could promote metastases. The finding that metastatic cells increase ROS production suggests the opposite. Overall, an elevated oxidative status has been associated with melanoma [42]. In fact, much effort has been expended to prevent or treat melanoma using antioxidants, aiming to counteract oxidative stress. The consequence of this redox-rebalance seems to be two-fold: (a) melanoma cells could behave less aggressively or even die; or (b) they could survive better after being disseminated into the circulating system or after drug treatment, thus resulting in metastasis promotion or further drug resistance [42]. We challenged the finding of Le Gal et al. [7] by giving Trolox (a lipophilic peroxyl radical scavenger) to metastatic B16-F10-bearing mice. Trolox (2.5 mg/kg × day) was administered i.p. (as in Usuki 2001) [43] for 10 days, starting 5 days after intraportal melanoma cells inoculation. On day 15, metastatic growth in the liver was measured. Control mice showed a 16.3 ± 4.2 % of the liver volume occupied by metastases, whereas the Trolox-treated mice showed a 9.0 ± 2.6 (*p* < 0.05, n = 10 mice per group). This a clear example showing that in vitro run migration/invasion tests do not reflect the complexity of the in vivo conditions.

Previously reported data suggest that in vivo administered NAC e.g., provides neuroprotection [44] or hepatoprotection [45] via the activation of the Nrf2-ARE signaling pathway, precisely the mechanism that leads to an increase in expression of different enzymes of the antioxidant defense system, i.e., those reported in Figure 4. In fact, in B16-F10 cells isolated from NAC-treated mice, nuclear translocation of Nrf2 increased as compared to controls (Figure 4C). Interestingly, it was argued that small GTPase RHOA signaling is responsible for the potential NAC-induced melanoma cell migration [7], which is in fact another Nrf2-dependent mechanism. As recently shown, e.g., by Ko et al. [46], Nrf2 regulates cell motility through RhoA–ROCK1 signaling in NSC lung cancer cells.

As shown in Figure 3, NAC administration increases the plasma levels of Cys and cystine. NAC administration also associates to a marked increase in the uptake of these two amino acids in metastatic melanoma cells (Table 1B). shRNA-induced inhibition of the main transport systems for Cys (ASC1) and cystine (xCT) shows that metastatic activity in NAC-treated mice decreases by approx. 50% if the cystine uptake is inhibited; whereas the inhibition of the Cys uptake does not significantly affect the effect of NAC (Table 2). Interestingly, expression of the xCT also appears regulated by Nrf2 in e.g., human breast cancer cells, in response to oxidative stress [47]. In addition, the shRNA-induced knockdown of the GCLC activity causes approx. an 80% decrease in the metastatic growth. These facts suggest a clear link between GSH, metastatic activity (see Carretero 1999) [3], and extracellular cystine as the main source of intracellular Cys for GSH synthesis. We also found that the NAC treatment-induced increase in cystine uptake through the xCT cystine/glutamate antiporter associates to an increase in the utilization of L-Gln by the metastatic cells (Table S1); thus providing an extra source of intracellular glutamate for the exchange. This metabolic adaptation can also fuel metastatic growth [48]. In fact, different cancer cells, i.e., melanoma cells, can become addicted to L-Gln [32]. Moreover, it has been demonstrated that selective inhibition of L-Gln metabolism can enhance antitumor T lymphocyte activity in e.g., triple-negative breast cancer [49]. All these facts suggest the potential implications of the NAC-induced metabolic adaptation in the metastatic cells.

Although in our experiments only the highest dose of NAC (given for a limited period of time) showed a clear increase in the metastatic spread (Figure 2), it is uncertain if lower doses given for a much longer period of time could be dangerous.

## 5. Conclusions

In conclusion, the present study demonstrates that high therapeutic doses of NAC can cause metastatic melanoma spread, even if the primary tumor has been surgically removed. Increased GSH levels and cystine uptake, and the switch to a higher L-Gln consumption in the metastatic cells, are the key underlying metabolic adaptations. NAC increases the generation of ROS in metastatic cells, therefore the mechanism does not imply an antioxidant effect. In fact, in vivo treatment with Trolox, a soluble analog of vitamin E and a direct antioxidant, decreases the metastatic growth. Therefore, some caution should be taken before administering Cys/cystine donors and/or GSH promoters in cancer-bearing patients, or in patients who have undergone surgical excision of a cancer with potential for metastatic spread.

## Figures and Tables

**Table 1:** Effect of NAC on the plasma levels of amino acid precursors for GSH synthesis in mice bearing B16-F10 xenografts (**A**), and on the GGT activity and cyst(e)ine uptake in B16-F10 melanoma cells isolated from growing xenografts (**B**). NAC (240 mg/kg) was administered orally to mice bearing B16-F10 xenografts starting 5 days after tumor inoculation. All measurements were performed in plasma or cells isolated 15 days after tumor inoculation. * Significantly different *p* < 0.05, comparing plasma levels of amino acids in B16-F10-bearing mice versus non-tumor-bearing mice (n = 5–6) (A); ^+^ Significantly different *p* < 0.01, comparing data obtained in metastatic cells isolated from mice treated NAC versus controls treated with vehicle (B) (n = 6).

**A**						
	**Amino Acid Concentration (μM) in Plasma**					
	**Non-Tumor-Bearing Mice**			**B16-F10-Bearing Mice**		
	**-**		**+NAC**	**-**		**+NAC**
Gln	543 ± 31		526 ± 39	416 ± 51 *		385 ± 32 *
Glu	132 ± 26		125 ± 17	87 ± 23 *		106 ± 21
Gly	327 ± 43		318 ± 40	306 ± 38		312± 36
Ser	169 ± 25		159 ± 37	152 ± 26		149 ± 24
Met	64 ± 14		59 ± 15	37 ± 10 *		30 ± 6 *
Cyst(e)ine	16 ± 3		184 ± 35 ^+^	21 ± 5		227 ± 42 ^+^
**B**						
		**GGT Activity and Cyst(e)ine Uptake in B16-F10 Cells**				
		**-**			**+NAC**	
GGT (mU/10^6^ cells)		35.5 ± 6.4			36.0 ± 5.3	
Cys uptake (nmol/mg protein × min)		4.3 ± 1.2			8.5 ± 2.3 ^+^	
Cystine uptake (nmol/mg protein × min)		27.2 ± 4.6			60.7 ± 9.7 ^+^	

**Table 2:** Effect of inhibition of Cys/cystine uptake or GSH synthesis on GSH levels and metastatic activity in B16-F10 cells. Cancer cells were transfected in vitro, as explained under Material and Methods, before their in vivo inoculation. B16-F10 cells were inoculated i.v. (portal vein). NAC (240 mg/kg) was administered orally for 10 days, starting 120 min after B16-F10 cells inoculation. All measurements were performed in the liver or in metastatic cells isolated from the liver 10 days after tumor inoculation. Results obtained using melanoma cells treated with scrambled RNA sequences were not significantly different from those displayed as non-treated controls. * Significantly different *p* < 0.01, comparing shRNA-treated cells versus controls; ^+^ Significantly different *p* < 0.01, comparing NAC-treated mice versus mice treated with vehicle (n = 9–10).

	Cys Uptake (nmol/mg prot. × min)		Cystine Uptake (nmol/mg prot. × min)		GSH (nmol/10^6^ cells)		Metastatic Activity (% of the Liver Volume Occupied by Metastases)	
Treatment	-	+NAC	-	+NAC	-	+NAC	-	+NAC
Control	4.0 ± 0.9	8.3 ± 1.7 ^+^	25.4 ± 3.8	62.3 ± 8.2 ^+^	26.2 ± 4.1	40.4 ± 5.0 ^+^	17.4 ± 3.6	30.2 ± 4.5 ^+^
Anti-ASC1-shRNA	0.7 ± 0.2 *	0.9 ± 0.3 *	26.2 ± 3.5	60.4 ± 7.7 ^+^	23.5 ± 3.3	38.3 ± 5.7 ^+^	15.9 ± 2.8	27.4 ± 3.6 ^+^
Anti-xCT-shRNA	3.9 ± 0.7	8.5 ± 2.0 ^+^	3.5 ± 0.6 *	6.4 ± 1.2 *^,^^+^	7.9 ± 1.3 *	9.6 ± 3.4 *	9.2 ± 1.5 *	16.7 ± 2.8 *^,^^+^
Anti-GCLC-shRNA	4.2 ± 1.0	8.5 ± 1.8 ^+^	27.9 ± 4.2	61.7 ± 6.9 ^+^	2.0 ± 0.5 *	3.5 ± 0.7 *^,^^+^	4.1 ± 0.7 *	5.8 ± 1.2 *

## Data Availability

The data presented in this study are available in the article and Supplementary Materials. Any additional information regarding the findings of this study is available from the corresponding authors upon reasonable request.

## Supplementary Materials

The following supporting information can be downloaded at: https://www.mdpi.com/article/10.3390/cancers14153614/s1. Figure S1: Western blot analysis of the effect of specific shRNAs on the levels of ASC1, xCT and GCLC in metastatic B16-F10 cells; Figure S2: Whole blots: (A) Figure 4C and (B) Figure S1; Table S1: Effect of NAC treatment on the rates of glucose and L-Gln utilization by the metastatic B16-F10 cells; Table S2: ROS generation in keratinocytes isolated from the skin of control non-tumor-bearing mice, of A2058 melanoma-bearing mice, and of mice where the A2058 melanoma was removed; Table S3: GGT activity, Cyst(e)ine uptake, GSH levels, metastatic activity, and ROS generation in B16-F10 metastatic cells isolated from the lung.
