# Supplementary material for: N-Acetylcysteine Promotes Metastatic Spread of Melanoma in Mice
Source: Cancers (Basel). 2022 Jul 25;14(15):3614. doi: 10.3390/cancers14153614 (PMC9331881; doi:10.3390/cancers14153614)
Supplement: Supplementary file 1 [file cancers-14-03614-s001.zip › cancers-1757406 - supplementary.pdf]

cancers

MDPI

Supplementary Materials

N-Acetylcysteine Promotes Metastatic Spread of Melanoma in Mice

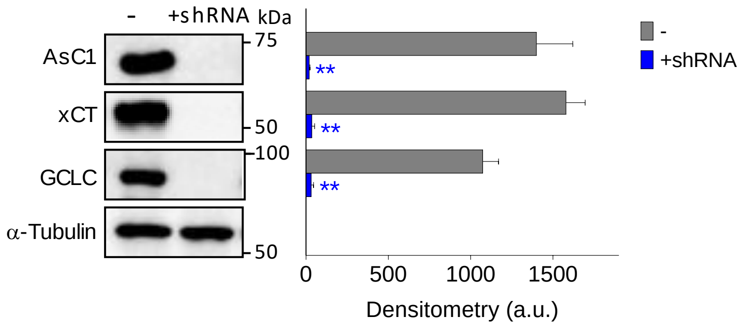

Figure S1. Western blot analysis of the effect of specific shRNAs on the levels of ASC1, xCT and GCLC in metastatic B16-F10 cells. Melanoma cells were inoculated, mice treated, and metastatic cells isolated as in Table 2. NAC treatment did not alter the effect of shRNAs (not shown). Densitometric analysis represents the mean values ± SD for three different mice per molecule and experimental condition (** $p < 0.01$, comparing shRNA-treated mice versus controls).

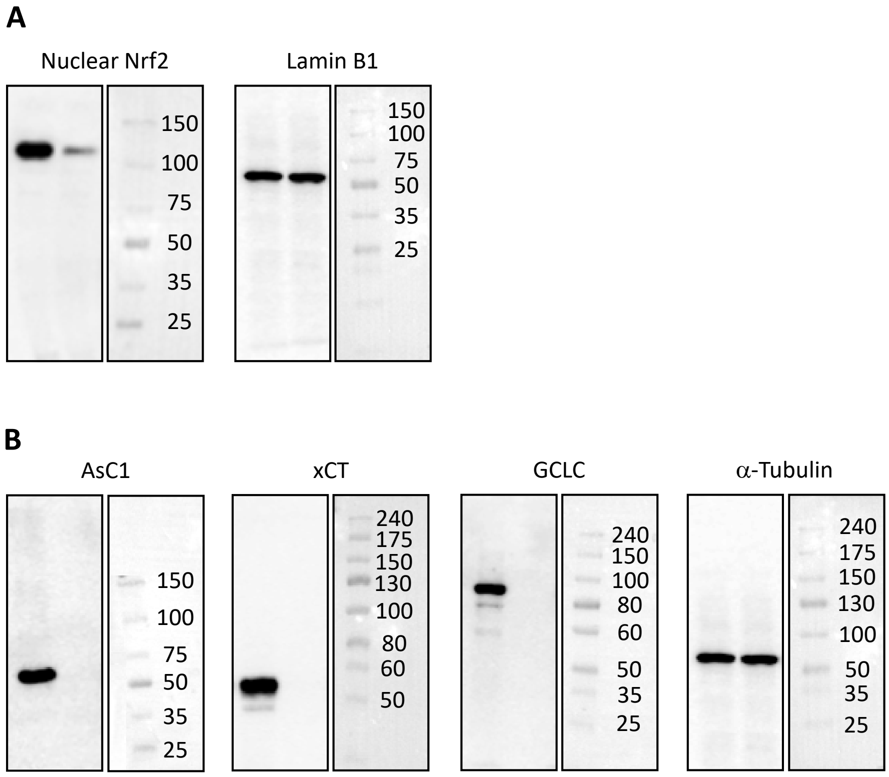

Figure S2. Whole blots: (**A**) Figure 4C and (**B**) Figure S1.

Table S1. Effect of NAC treatment on the rates of glucose and L-Gln utilization by the metastatic B16-F10 cells. Melanoma cells were inoculated, mice treated, and metastatic cells isolated as in Table 2. * Significantly different $p < 0.01$, comparing B16-F10 cells isolated from NAC-treated cells versus controls treated with vehicle (n = 5 in each case).

	nmol/10^6 Cells × min	
	-	+NAC
Glucose utilization	18.1 ± 2.3	12.5 ± 1.8 *
L-Gln utilization	29.4 ± 3.2	38.7 ± 2.1 *

Table S2. ROS generation in keratinocytes isolated from the skin of control non-tumor-bearing mice, of A2058 melanoma-bearing mice, and of mice where the A2058 melanoma was removed. Melanoma cells were inoculated and mice treated as in Table 2. * $p < 0.05$, ** $p < 0.01$ comparing all conditions versus control non-tumor-bearing mice; $^+$ $p < 0.05$ comparing NAC-treated mice versus controls (n = 5 in all cases).

	$O_2^{\cdot-}$ (nmol/10^6 Cells × min)		H_2O_2 (nmol/10^6 Cells × min)	
**Keratinocytes**	-	+NAC	-	+NAC
Non-tumor-bearing mice	0.20 ± 0.05	0.2 ± 0.1	0.5 ± 0.1	0.5 ± 0.2
Melanoma-bearing mice	0.4 ± 0.1 *	0.7 ± 0.2 **+	1.1 ± 0.3 **	1.8 ± 0.3 **+
Melanoma-removed mice	0.2 ± 0.1	0.2 ± 0.05	0.5 ± 0.1	0.6 ± 0.2

Table S3. GGT activity, Cyst(e)ine uptake, GSH levels, metastatic activity, and ROS generation in B16-F10 metastatic cells isolated from the lung. Melanoma cells were inoculated via the tail vein and mice were treated as in Table 2. * $p < 0.05$, ** $p < 0.01$ comparing NAC-treated mice versus controls (n = 7 in all cases).

	-	+NAC
GGT (mU/10^6 cells)	33.6 ± 4.7	34.5 ± 3.8
Cys uptake (nmol/mg protein × min)	4.0 ± 0.6	6.9 ± 1.2 **
Cystine uptake (nmol/mg protein × min)	24.1 ± 3.9	55.4 ± 12.4 **
GSH (nmol/10^6 cells)	20.2 ± 2.7	30.7 ± 3.5 **
Metastatic activity (% of lung volume occupied by metastases)	10.4 ± 2.4	20.5 ± 4.7 **
O_2 consumption (pmol/10^6 cells × min)	740 ± 106	1126 ± 129 **
$O_2^{\cdot-}$ generation (nmol/10^6 cells × min)	0.62 ± 0.11	1.07 ± 0.23 *
H_2O_2 generation (nmol/10^6 cells × min)	1.24 ± 0.33	2.07 ± 0.40 *
